# A longitudinal intervention to improve young children’s liking and consumption of new foods: findings from the Colorado LEAP study

**DOI:** 10.1186/s12966-019-0808-3

**Published:** 2019-06-03

**Authors:** Susan L. Johnson, Sarah M. Ryan, Miranda Kroehl, Kameron J. Moding, Richard E. Boles, Laura L. Bellows

**Affiliations:** 1 Department of Pediatrics, Section of Nutrition, University of Colorado Anschutz Medical Campus, School of Medicine, Aurora, CO USA; 2 Department of Biostatistics and Informatics, University of Colorado Anschutz Medical Campus, Colorado School of Public Health, Aurora, CO USA; 3 Department of Food Science and Human Nutrition, Colorado State University, Fort Collins, CO USA

**Keywords:** Preschooler, Eating behavior, Intervention, Longitudinal, Nutrition education, Vegetable intake, Neophobia

## Abstract

****Background**:**

Many interventions have been conducted to improve young children’s liking and consumption of new foods however their impacts on children’s consumption have been limited. Consistent evidence supports the use of repeated exposure to improve liking for new foods however longitudinal effects lasting greater than 6 months often have not been demonstrated. Here we report the eating-related findings of the Colorado Longitudinal Eating And Physical Activity (LEAP) Study, a multi-component intervention, delivered primarily in the school setting, which aimed to improve children’s liking and consumption of a target food via repeated exposure and positive experiential learning.

**Methods:**

Four sites in rural Colorado, each housing Head Start preschool programs, matched on state vital statistics for childhood obesity rates, (2 intervention and 2 control sites) took part in a quasi-experimental study design which included 4 time points (baseline, post-intervention, one-year [Y1] and two- year [Y2] follow ups). A total of 250 children and families were enrolled (*n* = 143 intervention and *n* = 107 control; 41% Hispanic and 69% low-income). A 12-week intervention, *Food Friends – Fun With New Foods®*, delivered by trained preschool teachers and which focuses on positive and repeated experiences with new foods, and a 5-month (1 unit/month) social marketing “booster program” was delivered in kindergarten (one-year follow up) and 1st grade (two-year follow up). Main outcome measures included change in children’s liking for new foods, analyzed by ordinal regression using generalized estimating equations, and change in weighed consumption of new foods over time, analyzed using a hierarchical mixed effects model.

****Results**:**

The intervention was delivered with good fidelity (87%). Both intervention and control groups demonstrated an increase in liking for the target food over time (*p* = 0.0001). The pattern of consumption of the target food was different, over time, for intervention and control groups (*p* < 0.005). In particular the change in intake between baseline and post-intervention was significantly greater in the intervention compared to the control group (*p* < 0.0001) though this pattern of change did not hold between baseline and Y2 follow up (*p* = 0.1144). Children in the intervention group who liked the target food consumed nearly double their baseline consumption at post-intervention (p < 0.0001;) and maintained this increase at Y2 follow up (p < 0.0001).

****Conclusions**:**

The *Food Friends* intervention, which utilized positive, repeated experiences with new foods, and was delivered with good fidelity by trained preschool teachers, found that larger improvements were observed in children’s eating behaviors than would be expected with developmentally-based changes in eating behaviors.

**Trial registration number:**

This trial is registered at ClinicalTrials.gov: NCT01937481.

Date registered: 09/09/2013; Retrospectively registered.

Date first participant registered: 09/15/2010.

## Background

Development of food preferences begins early in life and flavor exposure, via amniotic fluid, breastmilk and complementary foods, is important for the development of children’s food acceptance and dietary intake patterns [1]. The foods that children learn to like and eat prove to be important predictors of childhood health outcomes but also of future patterns of food preference [2–5]. It is therefore postulated that childhood is an important time to instill the acceptance and intake of healthy foods. Intrinsic factors associated with children’s food preferences include heritable factors (e.g., genetic predisposition to bitterness in foods) as well as trait-like factors (e.g., temperament and neophobia). Environmental factors also influence the development of food preferences [6–8]. Food availability [9, 10] as well as the opportunities and persistence with which children are offered to try new foods [11, 12], the feeding strategies utilized to engage children in trying them (e.g., pressuring vs. modeling; [13–15]), the social influences (peers, siblings, parents, and teachers; [16]), the setting (home and child care; [17, 18]), and the emotional valence of such opportunities all have been reported to impact children’s food preferences and consumption patterns [19, 20].

The most consistently reported mechanism to positively influence children’s acceptance of foods is that of repeated exposure [21, 22]. This strategy is grounded in Zajonc’s mere exposure theory [20] which suggests that individuals can develop preferences across a variety of domains if they are repeatedly exposed to a stimulus over time. For development of food preferences, several studies have demonstrated that if infants, toddlers, and children are exposed to the same food across multiple occasions, they can learn to accept that food [23–26]. The strategy of repeated exposure has demonstrated positive effects on both consumption and liking of novel and disliked foods [23, 27–29], including both vegetables and fruits [23, 27, 30]. However, one important aspect of mere exposure theory that has been commonly omitted in repeated exposure studies is the emotional valence in which the repetitions take place [31]; repetitions paired with positive emotions or experiences are likely to reinforce the development of food acceptance and preference, whereas repetitions paired with negative emotions or experiences are likely to reinforce negative taste preferences [6].

Despite the evidence that young children’s food preferences can change and include initially rejected foods, the majority of preschoolers consume fewer vegetables and whole grains, as well as more solid fat and added sugars, than the amounts recommended in the Dietary Guidelines for Americans [32, 33]. The shift toward less than ideal consumption patterns begins during early toddlerhood when young children’s diets begin to resemble adult diets [34, 35]. The proportion of young children (6–48 months) in the US who consume any vegetable during a 24-h period plateaus by 12 months of age (about 70% of children if including white potatoes and 60% of children if excluding white potatoes) and vegetables contribute little to young children’s total daily intakes in the United States [34, 35]. Children from racial/ethnic minority groups and children from families with limited resources and low-income communities in the US are most at risk of consuming less than adequate amounts of vegetables [35, 36]. Similar trends are noted across the developed world [37–39]. Vegetable consumption is particularly important and concerning given its role in reducing risk of chronic disease [40, 41].

Systematic reviews have summarized the number and magnitude of effects of home and community-based interventions aimed at increasing children’s liking and consumption of vegetables. The most robust effects have been noted for interventions which are theory-based [42] and which include repeated exposure, nonfood reward, and content delivered by researchers or external experts, or programs which include staff training related to feeding and introducing new foods [43, 44]. The mean effect of all interventions on preschooler vegetable consumption has been estimated to be a 29% increase (range from − 20 to 87%) in one review [17] and a 4.03 g increase (from a baseline of 7.7 g) in a Cochrane review of randomized controlled trials [43]. Two randomized controlled trials conducted in the preschool setting reported sustained effects of the intervention on vegetable consumption and these effects were ascertained at 3 and 12 months post-intervention [45, 46]. However, intervention effects are often measured by parent proxy report (e.g., survey or recall), rather than by objective measures, adding to the limitations of the generalizability of the findings. Among the conclusions and recommendations of the reviews were 1) a call for interventions which result in larger effect sizes; 2) follow up assessment periods which demonstrate longer duration of intervention effects; and 3) reduction of bias in intervention delivery and assessment [17, 43, 44].

The Colorado Longitudinal Eating And Physical activity (LEAP) Study utilized a social ecological and social marketing approach to explore the relationships among individual, family and environmental factors and children’s weight status over the course of early childhood (4–7 y of age; see [47] for more detailed information regarding study design). One of the primary aims of the study was to determine whether the previously established effectiveness of *The Food Friends- Fun with New Foods*® program [48, 49] on children’s willingness to try new foods could be sustained over time (two-year follow up from preschool into elementary school). A secondary aim was to determine whether improvements in target food hedonics (liking) would be associated with changes in children’s consumption of the target food during typical eating occasions in the school day.

Hypotheses specific to this part of our Colorado LEAP intervention included that the children in the intervention group, compared with those in the control group, would demonstrate: 1) greater increases in liking of the target food to which they would be repeatedly exposed; 2) greater intakes of the target food; and 3) that children who stated liking for the target food would consume more of it in test trials for consumption. We also hypothesized that these intervention effects would be sustained throughout the two-year follow up period of the study.

## Methods

### Overview

The methods, including details about the intervention content and delivery, as well as assessments utilized to assess the effectiveness of the intervention, have been previously detailed [47]. The Colorado LEAP Study was registered as a clinical trial retrospectively when authors realized the importance of this step (at ClinicalTrials.gov: NCT01937481). The study was registered during the mid-point of data collection prior to statistical analysis. A synopsis of each aspect of design, protocol, intervention and assessments is described below.

### Participants and study design

The Colorado LEAP study was a 3-year longitudinal study utilizing a controlled, quasi-experimental design in four rural Colorado communities – two mountain communities (tourism-driven economy; one intervention and one control) and two eastern plains (agricultural driven; one intervention and one control) communities. Intervention and control sites were matched on community level vital statistics (preschool obesity, childhood poverty rates and enrollment in federally sponsored healthcare for children [50]). Recruitment and delivery were conducted in Head Start/preschool sites with intervention and control groups matched at baseline on community demographics, Head Start Program Information Reports, and geographic location (rural plains, rural mountains). Preschools were first recruited and consent for site participation was received from center directors in Spring 2010. Participants were recruited by informational packets (English and Spanish), with an included consent form, that was sent home with their child. Additionally, participants were recruited through Parent Information events that were held at the preschools. Participants were enrolled between 2010 and 2012 and followed through 2015.

All families and children (except those who were not expected to progress to kindergarten the following year) were invited to participate in the study, however children with parent-confirmed developmental and intellectual disabilities and with food allergies were excluded from data analyses. This study was approved by the institutional review boards at Colorado State University and the University of Colorado Anschutz Medical Campus.

Intervention sites received *The Food Friends - Fun with New Foods®* program in preschool and ‘booster’ programming in kindergarten and 1st grade. Assessments were conducted pre- and post-intervention (Baseline in the Fall, Post-intervention in the Spring) in the preschool and in the spring in kindergarten (one-year follow up; Y1) and 1st grade (two-year follow up; Y2). The intervention and assessments are briefly described below. Written parental consent (returned via mail for some parents and in person for those who attended Parent Information nights) was obtained at baseline and child assent was obtained at each assessment time point.

### Intervention

**Fig. 1:**
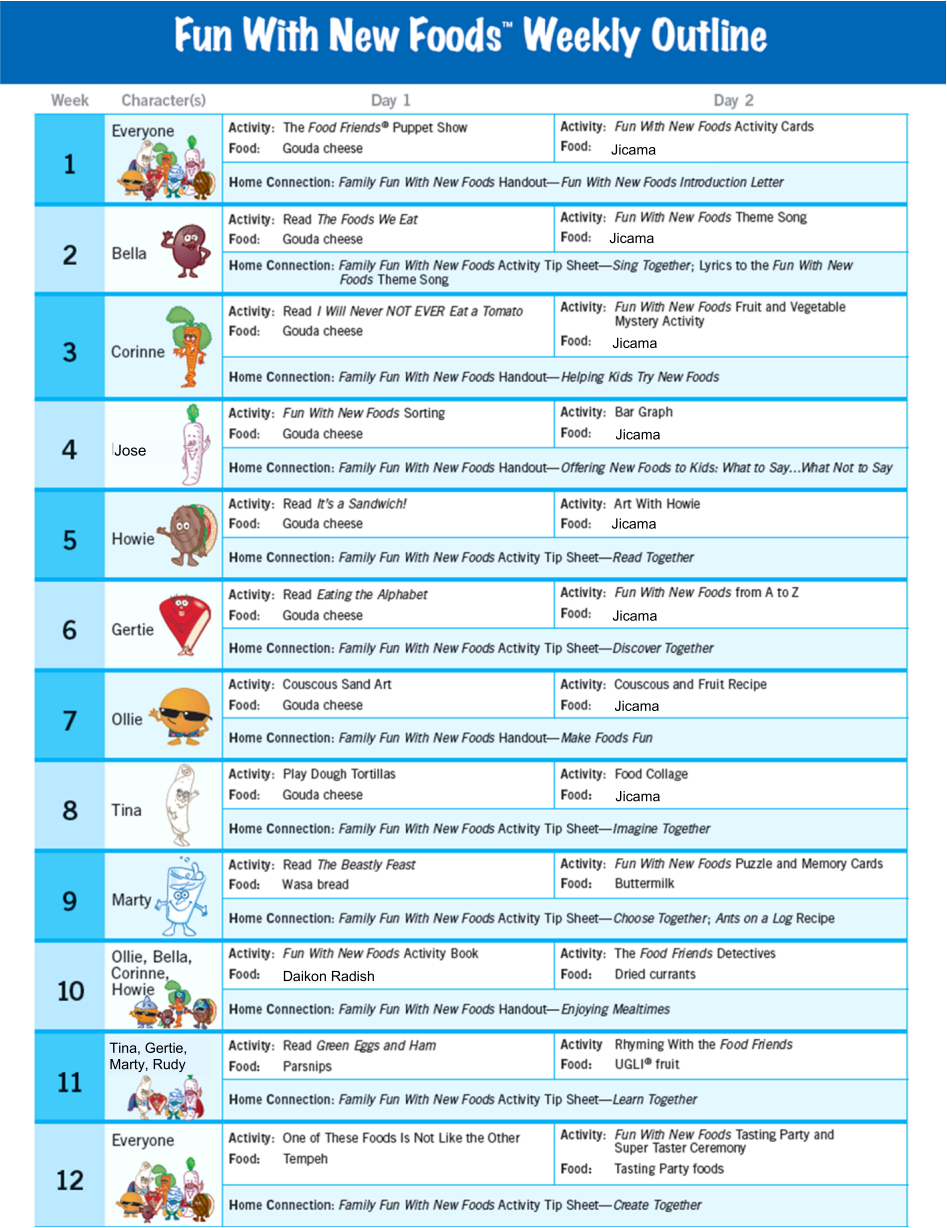
Table of lesson content for the *Fun with New Foods* Program

The *Food Friends - Fun With New Foods®* intervention program is a research-based preschool program designed to address childhood obesity by offering classroom experiences that promote the development of healthful eating behaviors; specifically improving young children’s willingness to try new foods [48, 49, 51]. The 12-week program was developed based upon constructs of social cognitive theory, tenets of social marketing, and is embedded within Bronfenbrenner’s social ecological framework [52]. Eight Food Friends characters are integral to the program and represent different food groups (e.g., Howie Hamburger and Jose Jicama) and are integrated into the program themes, activities, and materials. Children receive the program via their preschool classroom, delivered by trained teachers, through two activities per week for a total of 24 sessions (see Fig. 1 for lesson specifics). These 15- to 20-min fun and experiential nutrition activities promote school readiness via a puppet show, fruit and vegetable mystery bag, a tasting party, and puzzles, as examples, and thus support a positive valence during these learning experiences. Children also are presented with opportunities to try new foods two times per week; they are offered at least one novel food per week and repeatedly are offered one target food (jicama) one time per week for the first 8 weeks of the 12-week program (total of 8 exposures). Jicama was chosen based upon formative work within preschools and with parents to determine foods which most children had not tried but that were available in area stores for purchase [51]. In addition to the preschool curriculum, bilingual (English and Spanish) materials are sent to the home (Home Connection) to encourage parents to provide children with opportunities to learn about and try new foods at home [53]. Children in the control preschools received the school’s standard curriculum.

During follow up years (Y1 and Y2), a low intensity, 5-unit ‘booster’ program was provided, one time per month (15–20 min in duration), via the kindergarten and 1st grade classrooms, to support and sustain preschool behavior changes. Short activities conducted by trained nutrition educators served as reminders of program messages that were learned in preschool. The *Food Friends* Super Taster messages, in the form of banners and posters (e.g., social marketing), were displayed throughout the school environments. Activities mimicked those presented to children in preschool but were adapted for advanced grade levels. In addition, 5 packets of The Super Taster Club materials, including individualized child newsletters, bilingual parent newsletters, and educational enhancers (i.e., chef’s hat, spatula, recipe book) were mailed to the home monthly for 5 months.

### Participant incentives

Parents were compensated for their time in completing data collection measures ($20 US at T1 and T2, $40 US at T3 and T4). Schools were compensated for their implementation of the program or their support of study conduct ($500 US/school total). Teachers were given $50 US to complete surveys and to assist with communications with parents and distribution of data collection packets for parents.

### Assessments

Observational assessments of children’s willingness to try foods and consumption of novel foods were conducted by trained research staff during the school day at 4 time points: baseline, post-intervention, Y1 and Y2 follow ups.

### Demographic information

Parents completed a questionnaire at entry into the study that supplied demographic information including age, ethnicity, race, education, employment status, and income bracket. They completed this information at home and returned it in their child’s backpack or by prepaid mail envelopes.

### Teacher survey

*The Food Friends* Teacher Survey consisted of 11 questions seeking input on teachers’ favorite/least favorite program activities as well as ratings on children’s perceived interest in each of the 21 *Food Friends* activities. Teachers could also indicate if s/he ‘Did not do’ the activity with the class. Program fidelity was calculated as a percentage of total activities rated by teachers divided by total possible activities to complete.

### Children’s liking of novel foods

Children’s liking of novel foods was conducted via a modification of the Sullivan and Birch taste preference assessment [24]. Each child participated in the “Tasting Game” individually with one trained researcher. Each child was asked to taste 9 foods (jicama, garbanzo beans, grapefruit, Gouda cheese, couscous, spinach, salmon, beets, and pineapple). Several food attributes were considered when choosing this set of foods including: some familiar and some novel foods; sweet and savory foods; and representation across food groups (e.g., vegetable, protein, fruit, etc.); one was a target food to which children were repeatedly exposed via the *Food Friends* program (jicama). Children were asked to first taste and then rate each food as “Yummy”, “Just OK” “Yucky” using emoticons associated with each word to facilitate children’s understanding of the procedure. The ratings of the novel target food (jicama) for control and intervention groups, across the different assessment periods, will be reported here.

### Consumption of program target foods

Children’s consumption of two target foods, jicama (to which they were repeatedly exposed during the intervention) and edamame (a new food not encountered in the intervention), was tested at each of the 4 time points. The purpose was to determine intervention effects on children’s consumption of the repeatedly exposed target food (jicama) and consumption of an unexposed new food that was not encountered during the intervention (edamame). Portions of each food (65.0 ± 2.0 g) were pre-weighed (on an Ohaus digital scale to the nearest 0.1 g) and offered at lunchtime or snack time in children’s usual setting (i.e., in the classroom or in a lunchroom) at each time point (baseline, post-intervention, Y1 and Y2 follow ups). The target foods were offered as additional food items, served with other lunch or snack foods, at a typical meal or snack time. Each child’s containers of the target foods were individually post-weighed (to the nearest 0.1 g) and the amount consumed was calculated (to the nearest 0.1 g). As the focus of the assessment was the target foods, consumption of other foods at the meal or snack were not measured.

### Anthropometrics

Children’s weight and height were measured at each time point according the method of Lohman et al. [54] on a digital scale (Lifesource ProFit UC321; Milpitas, CA) to the nearest 0.05 kg and by portable stadiometer to the nearest 0.1 cm (Seca Corp, Hamburg, Germany) by trained research staff. Only baseline data are presented here as demographic characteristics. Body Mass Index (BMI; kg/m^2^) was calculated [55].

### Data analyses

Descriptive statistics were calculated for each variable. Ordinal regression using generalized estimating equations (GEE), assuming an independence working correlation structure for robust variance estimation, was used to examine differences in the liking ratings between intervention and control groups and changes over time. An interaction term between time and study group was evaluated to determine whether the change in liking ratings over time differed between intervention and control groups. A hierarchical mixed effects model, assuming type 1 autoregressive covariance structure and random intercept for study participants nested within study site, was used to estimate subject-specific target food consumption over time and to compare differences between intervention and control groups. Linear contrast statements were used to determine differences between time points and/or study group. An interaction term was evaluated to determine whether the effect of study group on consumption, over time, was dependent on liking rating. Both GEE and hierarchical mixed effects models assume data are missing at random, and thus children were included in the analyses if they had at least one observation across the four time points. Children who refused to taste a food during the liking ratings were not included in the ratings analyses as they had no basis upon which to accurately rate the food; however, they were counted as consuming 0 g for the consumption analyses. All models were adjusted for covariates, including child BMI (continuous), sex, ethnicity (White vs. non-Hispanic White), and parent income (< 100%, 100–185%, > 185% poverty line). Cohen’s d effect sizes were calculated for the comparisons of consumption between groups and between time points [56]. All analyses were performed using SAS version 9.4 (SAS Institute, Cary, NC).

## Results

**Fig. 2:**
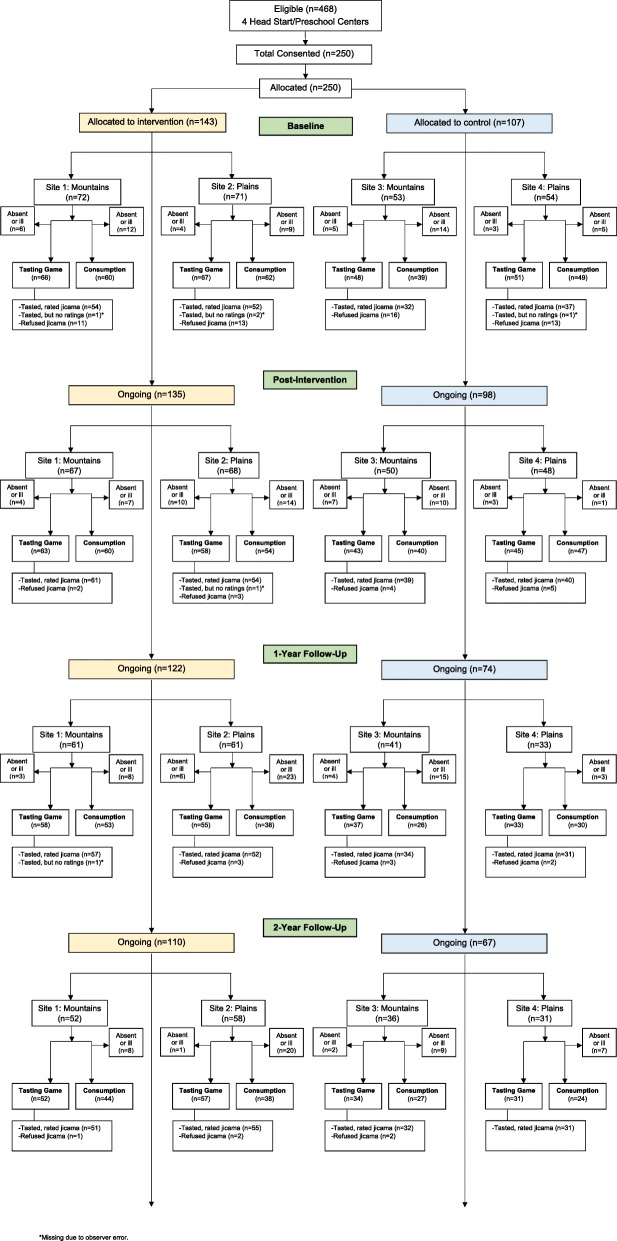
Consort diagram. Recruitment, enrollment and retention of participants by group and by site

Demographic characteristics of the children and their families are presented in Table 1. Children (*n* = 250; 54.4% female, 4.7 + 0.4 y) were mostly White (~ 41% Hispanic) and the majority were of normal weight status at baseline (29% overweight or obese). The majority of the parents were aged 30–49 years of age (57.3%), had engaged in some higher education (65.2%) and qualified for federal assistance programs (69.9%). Program fidelity was high with 87.0% (range: 71–100%) of activities completed in the classrooms (*n* = 13). Over the course of the 3-year study period, an average retention rate of ~ 70% of children was achieved (though not all children completed each assessment at each time point; see Fig. 2).

**Table 1:** Baseline demographic characteristics of the children participating in the Colorado LEAP Study

Characteristic		Total (*n* = 250)^1^	Intervention (*n* = 143)	Control (*n* = 107)
		X (SD)	X (SD)	X (SD)
Child Age		4.7 (0.4)	4.7 (0.3)	4.6 (0.4)
Child BMI		16.5 (2.4)	16.6 (2.6)	16.4 (2.1)
Child Sex Female Male		*n* (%) 136 (54.4) 114 (45.6)	*n* (%) 84 (58.7) 59 (41.3)	*n* (%) 52 (48.6) 55 (51.4)
Child Ethnicity	Hispanic/Latino	100 (40.7)	65 (45.5)	35 (34.0)
	Not Hispanic/Latino	146 (59.3)	78 (54.5)	68 (66.0)
Child Race	White Other^**2**^	147 (82.1) 32 (17.9)	80 (76.2) 25 (23.8)	67 (90.5) 7 (9.5)
Parent income/y^3^	Less than $41,000	114 (69.9)	72 (74.2)	42 (63.6)
	41,000 - $69,000	27 (16.6)	14 (14.4)	13 (19.7)
	More than $69,000	22 (13.5)	11 (11.3)	11 (16.7)
Parent age (y)	18–29	72 (40.4)	50 (46.7)	22 (31.0)
	30–49	102 (57.3)	53 (49.5)	49 (69.0)
	50–64	4 (2.2)	4 (3.7)	0 (0.0)
Parent education	Less than high school	20 (12.2)	13 (13.3)	7 (10.6)
	High school education	37 (22.6)	26 (26.5)	11 (16.7)
	College/some college education	107 (65.2)	59 (60.2)	48 (72.7)

^1^Variables have different sample sizes due to differences in caregiver reporting

^2^Other races include Black, American Indian/Alaska Native, Asian, Native Hawaiian/Pacific Islander, Mixed Race

^3^Less than $41,000 is a proxy for < 185% of poverty [73]

To conduct an analysis of missingness across intervention and control groups, we chose to analyze missingness for the primary outcome variable of jicama consumption. Of the 143 children in the intervention group, 21 (15%) were missing consumption data at baseline, 29 (20%) at post-intervention, 52 (36%) at one-year follow up, and 61 (43%) at two-year follow up; however, 139 (97%) children had consumption data at one or more time points. Of the 107 children in the control group, 19 (18%) were missing consumption data at baseline, 20 (19%) at post-intervention, 51 (48%) at one-year follow up, and 56 (52%) at two-year follow up; however, 101 (94%) children had consumption data at one or more time points. The percentage of missingness increased with time for both groups, however, missingness between the two groups was not statistically significantly different at any timepoint (*p* > 0.05 at all timepoints per chi-square test of independence).

### Liking ratings of the target food (jicama) over time

**Fig. 3:**
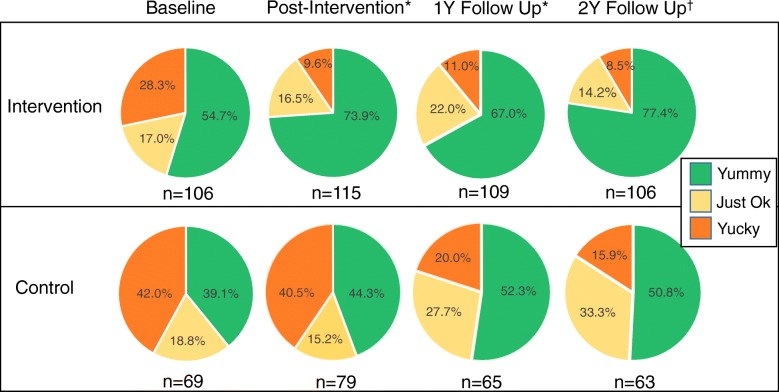
Proportion of children’s liking ratings of the target food (jicama) by the time point and by group (intervention and control). Per GEE analysis, there was a significant difference in liking ratings between study groups at baseline (*p* = 0.0002) and a significant overall change in liking ratings over time (p = 0.0002), however per interaction analysis the changes over time did not differ between study group (*p* = 0.1980). Statistically significant differences between follow up times and baseline are indicated by * (*p* < 0.05) or † (*p* < 0.001). Y1 = year-one follow up; Y2 = year-two follow up

Across both intervention and control groups, ratings changed significantly from baseline to each follow up interval (*p* = 0.0002), with overall increases in ‘yummy’ ratings and decreases in ‘yucky’ ratings (Fig. 3). There was a statistically significant difference in likings ratings between study groups at baseline; the intervention group had more “yummy” ratings compared to the control group (54.7 vs. 39.1% rated as yummy, 17.0 vs. 18.8% for just ok, 28.3 vs. 42.0% for yucky, for intervention and controls, respectively; p = 0.0002). Additionally, after repeated exposure to jicama during the classroom program, the intervention group demonstrated a 19.2% increase in children classifying jicama as ‘yummy’ from baseline to post-intervention, compared to an 5.2% increase in the control group. This increase in the proportion reporting jicama as ‘yummy’ was sustained over time in the intervention group compared to the control group (22.7% vs. 11.7% between baseline to Y2 for intervention and control, respectively). However, while the intervention group saw numerically larger increases in liking ratings over time as compared to the controls, the differences between intervention and control did not reach statistical significance, per the interaction analysis (interaction term *p* = 0.1980; Fig. 3).

### Children’s consumption of the target foods over time

**Fig. 4:**
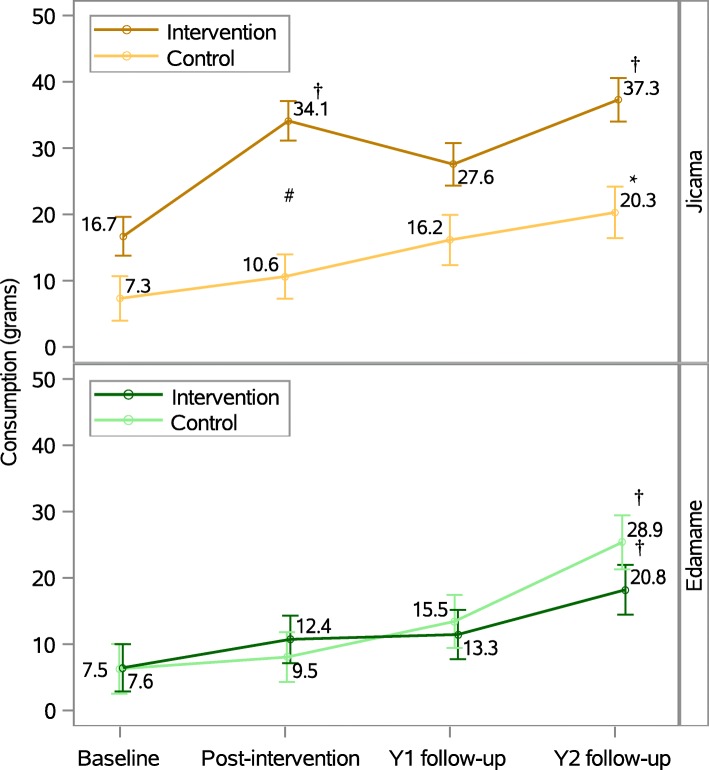
Change in consumption (g) for jicama (Fig. A in brown) and edamame (Fig. B in green), adjusted for sex, ethnicity, BMI, and parent income. Bars represent standard errors obtained using linear contrast statements from a hierarchical linear mixed model. To note statistically significant differences between follow up times and baseline, a * (*p* < 0.001) or † (*p* < 0.0001) is used. # indicates a significant difference in the change from baseline to post-intervention between intervention and control groups (p < 0.05). Y1 = year-one follow up; Y2 = year-two follow up

There was a significant difference in the pattern of consumption of jicama over time between intervention and control groups (*p* < 0.005; Fig. 4). Children in the intervention group ate significantly more jicama post-intervention (*p* < 0.0001, d = 0.68) and at the Y2 follow up (*p* < 0.0001, d = 0.86; Table 2) compared to consumption at baseline. No difference was noted in consumption between baseline and post-intervention for the control group (*p* = 0.3094, d = 0.26) but consumption significantly increased between baseline and the Y2 follow up (*p* = 0.0008, d = 0.74). The change in intake between baseline and post-intervention was significantly greater in the intervention compared to the control group (p = 0.0008, d = 0.67); however, the change in intake between groups was not significant between baseline and Y2 follow up (*p* = 0.1144; d = 0.35).

**Table 2:** Children’s consumption of target foods at baseline, post-intervention and at 2-year follow up

	Group	Baseline	Post-intervention	Y2 follow up	Δ Intake Post-intervention – Baseline		Δ Intake Y2 follow up – Baseline	
		Mean (95% CI)	Mean (95% CI)	Mean (95% CI)	Mean (95% CI)	*P*-value	Mean (95% CI)	*P*-value
Jicama	Intervention	16.7^1^ (10.9, 22.5)	34.1 (28.2, 40.0)	37.3 (30.8, 43.8)	17.4 (12.3, 22.5)	<.0001	20.6 (14.8, 26.4)	<.0001
	Control	7.3 (0.7, 14.0)	10.6 (4.1, 17.2)	20.3 (12.6, 28.0)	3.3 (− 3.1, 9.7)	0.3094	13.0 (5.4, 20.5)	0.0008
	Difference	9.4 (1.6, 17.2)	23.5 (15.6, 31.4)	17.0 (7.7, 26.3)	14.1 (5.9, 22.3)	0.0008	7.6 (−1.9, 17.1)	0.1144
Edamame	Intervention	7.6 (−0.2, 15.5)	12.4 (4.5, 20.4)	20.8 (12.6, 29.1)	4.8 (0.5, 9.1)	0.0280	13.2 (8.0, 18.4)	<.0001
	Control	7.5 (−0.9, 15.8)	9.5 (1.2, 17.7)	28.9 (19.9, 37.9)	2.0 (−3.4, 7.4)	0.4652	21.4 (14.6, 28.2)	<.0001
	Difference	0.2 (−10.8, 11.2)	3.0 (−8.1, 14.0)	−8.0 (− 19.9, 3.8)	2.8 (−4.1, 9.7)	0.4208	−8.2 (− 16.8, 0.4)	0.061

^1^All units are in grams

^2^P-values obtained using linear contrast statements from a hierarchical linear mixed model; the significance level was set at *p* < 0.05

For edamame, there was a significant increase in consumption over time across both groups (*p* < 0.0001). However, there was no difference in the pattern of consumption of edamame between intervention and control groups (*p* = 0.061, d = − 0.39; Fig. 4).

### Relationship between children’s liking ratings and their consumption of the target food over time

**Fig. 5:**
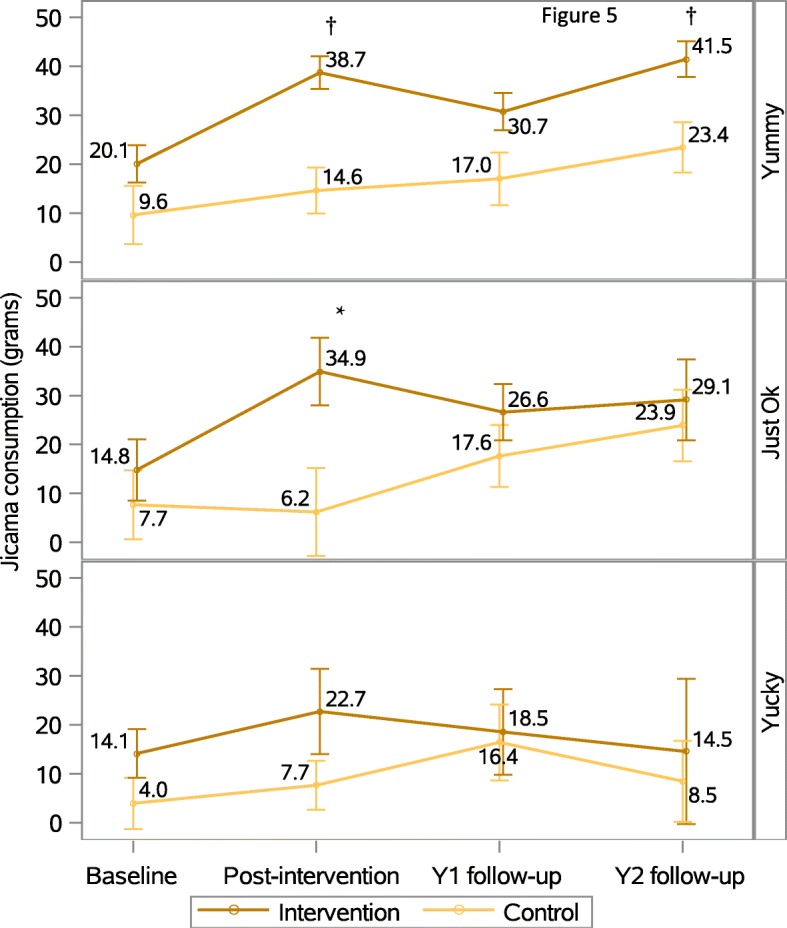
Change in jicama consumption for children rating food as ‘yummy’ (top), ‘just OK’ (middle), and ‘yucky’ (bottom), adjusted for gender, ethnicity, BMI, and parent income. Bars represent standard errors obtained using linear contrast statements from a hierarchical linear mixed model. The liking ratings reflect those specified by children at each time point. To note statistically significant differences between follow up times and baseline, a * (*p* < 0.05) or † (*p* < 0.0001) is used. Y1 = year-one follow up; Y2 = year-two follow up

Children in the intervention group who classified jicama as ‘yummy’ consumed nearly double their baseline consumption at post-intervention (consuming ~ 2/3 serving; *p* < 0.0001, d = 0.64; Table 3) and maintained this increase at Y2 follow up (*p* < 0.0001, d = 0.79). Children in the control group who classified jicama as ‘yummy’ did not significantly increase their intake at post-intervention nor at Y2 follow up, though their increase in intake at Y2 follow up approached significance (~ 1/3 serving, *p* = 0.0505, d = 0.30). When comparing the increases in intake from baseline to post-intervention or Y2 follow up for children who rated jicama as ‘yummy’, the difference in the increase between the intervention and control groups did not reach significance (Fig. 5).

**Table 3:** Children’s consumption of jicama at baseline, post-intervention and at 2-year follow up by liking group

Food rating	Group	Baseline	Post- intervention	Y2 follow up	Δ Intake Post-intervention - Baseline		Δ Intake Y2 follow up - Baseline	
		Mean (95% CI)	Mean (95% CI)	Mean (95% CI)	Mean (95% CI)	P-value^2^	Mean (95% CI)	P-value
Yummy	Intervention	20.1^1^ (12.5, 27.6)	38.7 (32.1, 45.3)	41.5 (34.3, 48.6)	18.6 (11.0, 26.3)	<.0001	21.4 (13.4, 29.5)	<.0001
	Control	9.6 (−2.1, 21.4)	14.6 (5.4, 23.8)	23.4 (13.3, 33.5)	5.0 (−7.3, 17.3)	0.4247	13.8 (−0.0, 27.6)	0.0505
	Intervention-Control	10.4 (−3.0, 23.9)	24.1 (13.4, 34.8)	18.0 (6.3, 29.8)	13.7 (−0.8, 28.1)	0.0646	7.6 (−8.4, 23.6)	0.3503
Just OK	Intervention	14.8 (2.5, 27.1)	34.9 (21.3, 48.5)	29.1 (12.9, 45.4)	20.1 (3.2, 37.1)	0.0203	14.3 (−5.0, 33.7)	0.1457
	Control	7.7 (−6.2, 21.6)	6.2 (−11.5, 23.9)	23.9 (9.4, 38.3)	− 1.5 (−22.4, 19.4)	0.8884	16.2 (− 2.7, 35.0)	0.0919
	Intervention-Control	7.1 (−10.9, 25.1)	28.7 (6.7, 50.7)	5.2 (−16.1, 26.6)	21.6 (−5.3, 48.5)	0.1144	−1.9 (−28.8, 25.1)	0.8922
Yucky	Intervention	14.1 (4.3, 24.0)	22.7 (5.6, 39.9)	14.5 (−14.7, 43.8)	8.6 (−9.7, 26.8)	0.3553	0.4 (−30.0, 30.8)	0.9799
	Control	4.0 (−6.4, 14.3)	7.7 (−2.2, 17.6)	8.5 (−7.8, 24.8)	3.7 (−8.5, 15.9)	0.5510	4.5 (−13.7, 22.7)	0.6246
	Intervention-Control	10.2 (−3.5, 23.9)	15.1 (−4.5, 34.6)	6.0 (−27.3, 39.4)	4.9 (−17.1, 26.8)	0.6612	−4.1 (−39.5, 31.3)	0.8183

^1^All units are in grams

^2^*P*-values obtained using linear contrast statements from a hierarchical linear mixed model; the significance level was set at *p*< 0.05

Children in the intervention group who classified jicama as ‘just ok’ ate significantly more jicama post-intervention (*p* = 0.0203, d = 1.0) compared to baseline, however, this effect was not sustained at the Y2 follow up (*p* = 0.1457, d = 0.9; Fig. 5). No significant effects were noted for children in the control group who rated jicama as ‘just ok.’ There were no significant patterns seen in children who classified jicama as ‘yucky’ (Fig. 5).

## Discussion

### Model for intervention effects

The *Food Friends* intervention, delivered via the classroom and based upon mere exposure theory, was successful in increasing consumption of a novel target food in the intervention group, compared to a control group. Most importantly, the increase in children’s consumption of the target food in the intervention group was sustained two years post-intervention. Differences in consumption between intervention and control groups were statistically significant only at post-intervention due to an increase in consumption by children in the control group over the two-year period (rather than a decline over the two year period in intervention group’s consumption).

**Fig. 6:**
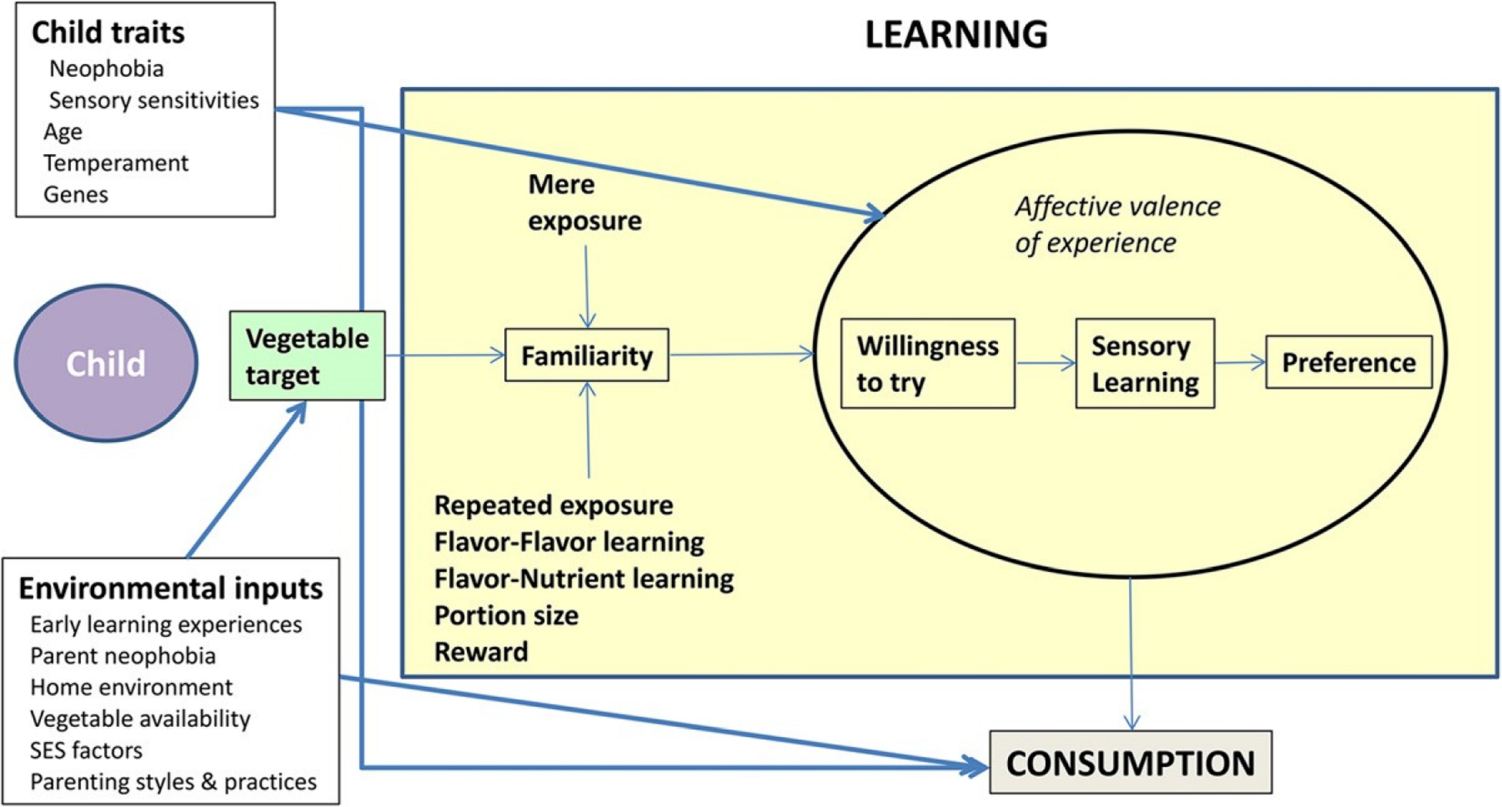
A 2-stage model of influences on the development of children’s vegetable preferences and consumption. Stage 1 reflects influences on children’s willingness to try vegetables. Stage 2 considers inputs on children’s vegetable consumption. SES = Socioeconomic Status. Original figure from: Johnson SL. Developmental and Environmental Influences on Young Children’s Vegetable Preferences and Consumption. Advances in Nutrition. 2016;7:220S–31S. Permission to reproduce has been requested

Consistent with our previously published model that articulated how increases in novel food consumption are produced via repeated exposure ([6]; Fig. 6), children who rated jicama more favorably demonstrated increases in consumption. The change in consumption, according to liking rating, was most striking for children in the intervention group: children who rated the target food as “yummy” demonstrated significant increases in consumption immediately following the intervention and sustained this increase two years post-intervention. That the intervention produced changes in jicama intake is also supported by the finding that children in the intervention who rated jicama as “just ok” consumed more post-intervention compared to their baseline consumption. This aligns with the model in that changes in preference co-occur (or perhaps precede) changes in children’s intake and that gradual shifts in liking are sufficient to produce some change in consumption. An alternative interpretation of these findings is that children who liked the taste of the target food were more influenced by the intervention.

Consumption of the target food by children in the control group also improved, but not to the same extent as in the intervention group. The increase in intake of the target food (jicama) by both intervention and control groups suggests that in addition to the intervention effects (i.e., at post-intervention), there appears to be a developmental shift in children’s ratings of the target food as well as their willingness to consume it (i.e., at Y2 follow up). These findings are in alignment with the literature which suggests that children’s neophobia dissipates (albeit, sometimes slowly) after 5 years of age [57]. Thus, while increases in willingness to consume new foods can occur by development alone, interventions could result in earlier benefits related to consumption.

In our study, it does not appear that repeated exposure to one target novel food generalized to willingness to consume a different novel food. Small increases in consumption of a nonprogram novel food occurred (edamame), and an increase from baseline was noted for the intervention group at the post-intervention time point. However, by two-year follow up, there were no between-groups differences in children’s consumption of edamame. Further, edamame consumption in the intervention group was about ½ the amount noted for consumption of the exposed program food (jicama). Again, this supports that, on average, children are more likely to increase their consumption of novel foods after the preschool period however such increases may be smaller and perhaps less likely to be appreciated by caregivers.

### Processes underlying intervention effects

Reviews of interventions targeting preschooler fruit and vegetable intakes have concluded that the most effective interventions are based upon a theoretical framework and include staff training to implement interventions in the classroom [43], rather than researcher-implemented interventions [42]. Both the *Food Friends* preschool and booster programs were grounded in Social Cognitive Theory (i.e., reciprocal determinism, behavioral capability, self-efficacy), utilized tenets of Social Marketing (i.e., marketing mix, audience segmentation, competition [51]) and were embedded within the social ecological model. This theoretical foundation was also applied to teacher training components. Preschool teachers participated in a two-hour training session and elementary teachers participated in a one-hour training covering core program concepts (i.e. picky eating, feeding young children), *Food Friends* intervention components and implementation strategies.

Behavior change strategies which have been successfully employed and have consistently resulted in improvements in children’s intake include repeated exposure [21, 58–61], use of nonfood rewards [25], adult and peer modeling [62, 63], or some combination thereof [59, 64]. Food adaptations (i.e., vegetables with dips, changing the visual presentation of the food, or flavor-flavor/flavor nutrient learning) for the most part have not produced additional effects beyond those of repeated exposure [23, 58, 65] with the exception of presenting bitter vegetables paired with dips [66]. Whether by masking of the bitter taste of the vegetables (i.e., mixture suppression, [67, 68] or from the perceived enjoyment of “dipping” the vegetables, children’s consumption of the target vegetable increased when paired with dips.

In the previously referred to studies, the size of the intervention effect achieved was usually modest. In a recent Cochrane review published by Hodder and colleagues [43], a meta-analysis revealed that child-feeding interventions (including those utilizing repeated exposure) resulted in an average 4.03 g increase in children’s consumption of a target vegetable, up to 12 months post-intervention. This Cochrane review also noted that the estimate was based upon very low-quality evidence (due to study limitations, inconsistency of effects, imprecision of measurement, and bias). Conclusions of the review called for 1) interventions which result in larger effect sizes, 2) longer intervals post-intervention for follow up assessment, and 3) study designs that reduce bias associated with self-report measures provided by adult caregivers. The present study resulted in a larger intervention effect (difference of 14 g consumed at one eating occasion and moderate to large within group effect sizes), the maintenance of this effect for 2 years post-intervention, and data collection obtained by observed measures in the early childhood setting in which the intervention was conducted.

### Advancements of the *Food Friends* intervention

Distinguishing features of the *Food Friends* intervention that we believe may relate to our positive outcomes include that the program is conducted and assessed within the early childhood education setting by trained early childhood educators and that it promotes learning experientially both through repeated exposure but also through positive interactions with food and eating (i.e., through imaginative characters and game play). Zajonc [31], who originally developed mere exposure theory, noted that the valence of the experience is an important element of the model for conditioning liking via repeated exposure (Fig. 4). Our findings suggest that engagement through fun activities which promote learning in a positive environment (e.g., use of characters and interactive games) may be an important factor in increasing young children’s willingness to try and in improving their preference for novel foods. We suggest that positive valence may be responsible for the magnitude of difference in consumption and liking reported for the intervention and control groups. By way of contrast, it has been clearly demonstrated the negative experiences (e.g., pressure to eat, controlling feeding practices) are associated with child neophobia and picky eating [69–71]. Here, we make a case for the influence of positive emotional tone for producing desired changes in children’s eating behaviors.

### Limitations of the study

Some important limitations of our study include that it was not feasible for children and schools to be randomized to treatment and control groups and thus there could be situational factors (location, classroom, etc.) that introduce bias in our conduct of the intervention. Further, while staff who administered data collection protocols were partially masked to the identity of treatment and control groups (i.e., no explicit indication of treatment group was conveyed), they were not fully blinded given that social marketing materials were visible in the schools when follow up data collection was being conducted. We note that changes in children’s liking ratings and their consumption of the jicama and edamame could have been influenced by the exposure that was a result of the assessment itself, though four exposures over three years is a lower level of exposure than the literature suggests is necessary to produce increases in preference [24]. Of note, differences existed in our outcomes at baseline, by group. These baseline differences could have been impacted by the children’s exposure to the target foods (either at the community or home level) in these rural locales pre-intervention. It is also possible that prior to intervention, the existing curricula in the preschools could have varied in how and the extent to which children were engaged in trying new foods. We recognized there was a difference and included a random intercept for site in our analyses. We included teacher self-report of fidelity to intervention delivery but did not collect direct observations of fidelity. Therefore, information regarding dose and completeness of intervention delivery may be subject to bias. Each of these factors had the potential to influence our intervention outcomes. As might be expected with a longitudinal study, we experienced loss to follow up, though between 63 and 75% of the total sample (slightly more children participated in the tasting task than in the consumption task) was retained at 2-y follow up for the measures reported here. Lastly, while the results presented here are focused on activities in the child care center, we endorse that the home environment, including parenting behaviors and food availability and accessibility [72], are important influences on child behaviors, each of which has been examined individually in previous research. In future work, we will examine the collective impact of child care and home environments on child behaviors to integrate multiple spheres of influence from a social ecological lens.

## Conclusions

Despite these limitations of the study, our findings are responsive to calls for interventions that create greater positive change in children’s eating behavior and that are implemented to lessen measurement bias in that observed measures involving tasting and consumption of target foods was undertaken and data collection staff were at least partially masked to the identity of treatment and control groups. The current data confirm that *The Food Friends* preschool intervention program results in improvements of children’s consumption of novel foods. Additionally, our findings highlight that experiential preschool nutrition education programs which focus on positive repeated exposure to new foods yield improvements in children’s eating, possibly greater than natural improvements in child eating behaviors related to vegetable consumption that develop over time.

## Abbreviations

BMI: Body Mass Index
Colorado LEAP Study: Colorado Longitudinal Eating And Physical activity Study
GEE: Generalized Estimating Eqs.
Y1: one-year follow up
Y2: two-year follow up

## Authors’ information

This project was conducted as a collaboration between Colorado State University (LLB, PI) and the University of Colorado Anschutz Medical Campus (SLJ, site PI). From its initiation, RB was a co-investigator and later, KJM, MK, and SR were added as authors to conduct longitudinal statistical analyses and to participate in presentation of the findings.
